# Injury severity-based discrepancies in severe trauma survival improvement

**DOI:** 10.1007/s00068-025-02946-z

**Published:** 2025-08-28

**Authors:** Benjamin Maurice Hardy, Natalie Enninghorst, Zsolt Janos Balogh

**Affiliations:** 1 Department of Traumatology, John Hunter Hospital, Newcastle, NSW Australia; 2 Discipline of Surgery, School of Medicine and Public Health, University of Newcastle, Newcastle, NSW Australia; 3 Injury and Trauma Research Program, Hunter Medical Research Institute, Newcastle, NSW Australia

**Keywords:** Polytrauma, Trauma, Multiple trauma, Trauma centers, Trauma system

## Abstract

**Purpose:**

Current trauma outcome reporting via registries captures nearly all trauma patients at risk of death. Most of these patients have milder injuries and they considerably outnumber the most critically injured patients. A change in outcomes of the whole group, may not be shared equally among all patients. Change or stasis in outcomes of the most severely injured could be masked by this bulk of more mildly injured trauma patients. We sought to examine the contemporary trends at reducing in-hospital mortality at highest injury severity patient group and hypothesized that mortality improvements are similar to all trauma patients included in a state trauma registry.

**Methods:**

All patients with an Injury Severity Score of 13–75 in a state of 8 million people, were included over a 10-year period ending in December 2021. Patients, and their demographic, injury severity, and outcome data were retrieved from the state’s trauma registry. Data were analysed using multiple logistic regression, dividing injury severity groups into the registry reported ISS ranges of 13–39 and 40–75, and the 40–75 group further divided into 40–49, 50–94, and 75.

**Results:**

27,862 patients who were admitted into the seven level 1 trauma centres of the state met inclusion criteria. The in-hospital mortality significantly decreased over the study period [OR: 0.976 (95%CI: 0.962–0.990)]. The ISS40-75 [*n* = 1,111 (4%)] patients’ mortality did not change during this decade [OR: 1.005 (95%CI: 0.963–1.049)]. On subgroup analysis, the ISS40-49 had worsening mortality [OR: 1.079 (95%CI: 1.012–1.150)], the ISS50-74 group’s mortality improved [OR: 0.913 (95%CI: 0.850–0.980)] and ISS75 group had no change [OR: 1.049 (95%CI: 0.878–1.252)].

**Conclusion:**

The overall improvement in severely injured patients’ mortality does not translate to the most critically injured ones. Further to this, the highest injury severity patients are not a homogenous group based on mortality improvement. Our analysis has identified the ISS 40–49 population for targeted quality improvement.

## Introduction

Trauma remains one of the leading causes of loss of life, worldwide [1]. The systematic measurement of trauma outcomes, and the public reporting of these, has become a global standard [2, 3]. Most published reporting of trauma mortality demonstrates improvement over time [4, 5]. Despite its obvious limitations – namely the broad range of functional outcomes in survivors – mortality is the most frequent outcome routinely reported globally, and one of the few outcomes that can be reliably collected retrospectively [6].

Severe injuries are rare when compared with moderate injuries [7]. Mortality is far more likely in those with severe injury [7]. Some trauma registries impose a minimum injury severity, to focus their collection on major trauma [8, 9]. One method is to use an injury severity score threshold [10]. The injury severity score (ISS) is an anatomically based scoring calculation and produces numerical score between 0 and 75, with 0 being no injury, and 75 representing injuries considered unsurvivable [7]. The ISS is composed of the three worst body region scores calculated using the Abbreviated Injury Scale (AIS), itself a 0–6 scale [11]. Other anatomic and anatomic plus physiological classifications exist as the Newcastle and Berlin definitions of polytrauma. These definitions use AIS of 3 or higher in two body regions, and in the case of the Berlin criteria, add a requirement for physiological [12, 13]. The polytrauma definitions define a smaller group of patients with a higher mortality but will not include patients with severe single system injury.

Conventionally ISS16-75 is used as a minimum threshold for major trauma [14], and in some jurisdictions, ISS13-75 is used [10]. Other thresholds are practical in larger data sets, with far higher minimum severities such as ISS50-75 [2]. In a previous single-centre study, much of the overall improvement in the ISS > 15 group was driven by patients not in in the ISS50-75 group [15]. The same centre contributes to a trauma registry that classifies ISS 40–75 as “critical injury” [16]. A variety of ISS thresholds (ISS40-,41-,42-,46-,50-, and 51–75) have been used over the history of the ISS with little standardization, and limited reporting [17]. We hypothesized that the mortality improvement in a large statewide registry would be distributed equally across all ISS subgroups, and particularly, the ISS 40–75 group reported in the registry.

Methods.

Study Population and Inclusion Criteria.

The study was conducted in New South Wales, Australia, state of eight million people with a size of 801,150 square km. The capital city contains 66% of the state’s population. There are seven major trauma centres all of which are based on the coastline and are supported by a centrally coordinated aeromedical retrieval service with helicopter and fixed wing assets. All trauma centres in this mature trauma system are verified by the Australia and New Zealand Trauma Care Verification Program of the Royal Australasian College of Surgeons adapted from the American College of Surgeons verification system [18]. The state contains a variety of light and heavy industries, and a considerable burden of motor vehicle transport due to the considerable distances in the state.

All trauma centres prospectively collect data on patients in a state trauma registry. ISS scores are calculated using the 2008 revision of the Abbreviated Injury Scale. The registry includes all patients who were admitted to a trauma service with an ISS > 12, or were admitted to an intensive care unit, or died following injury. Data were extracted from the registry for the period between January 1 st, 2012, and December 31 st, 2021. Age, sex, length of stay, Injury Severity Score, and mortality data were extracted. Injury severity score thresholds were defined as 13–39 and 40–75 as defined in the registry’s annual report [16]. The 40–75 group was further divided into 40–49, 50–75, and 75 based on the thresholds defined by Rozenfeld et al. [7]. The project was approved by the ethics committee (approval number 2022/ETH01099) and was conducted in accordance with the World Medical Association Declaration of Helsinki [19].

### Data analysis

Data was analysed using Stata 18 (StataCorp. 2023. Stata Statistical Software: Release 18. College Station, TX: StataCorp LP.). Continuous data are presented as median (IQR) and categorical data as counts and proportions. Comparisons between continuous variables was with the Kruskal-Wallis test. Proportions were compared using Fisher’s exact test. Binary logistic regressions were used to test for change in mortality over time; these were reported as odds ratios and 95% confidence intervals There was no adjustment for age, sex, or injury type. Date of injury was divided into calendar year and inserted into the model as an ordinal variable. Statistical significance was set at 5%. The STROBE cohort checklist was used when drafting the manuscript [20].

## Results

There were 27,862 patients admitted to major trauma centres, with an ISS13-75 collected between 2012 and 2021. There were 1,111 (4.0%) critically injured, ISS40-75 patients in the sample. This group was outnumbered 24:1 by the ISS13-39 group and had a mortality of 40.0%. The groups were not comparable in age; the less severely injured ISS13-39 patients were significantly older than the ISS40-75 patients (Table 1).

**Table 1:** Demographics and outcome by registry ISS grouping

	Registry ISS grouping			
	13–39	40–75	Total	*p*-value
Patients, n (%)	26,751 (96.0%)	1,111 (4.0%)	27,862 (100.0%)	
Age (years), median (IQR)	52 (30–73)	36 (21–57)	52 (30–72)	< 0.001
Sex, n (%)				
Female	7,687 (29%)	290 (26%)	7,977 (29%)	0.057
Hospital Length of Stay (days, median, IQR)	7 (4–15)	15 (1–43)	8 (3–16)	
ICU Length of Stay (days, median, IQR)	0 (0–2)	5 (1–14)	0 (0–2)	
Mortality, n (%)	2,291 (8.6%)	444 (40.0%)	2,735 (9.8%)	
Logistic regression for mortality by registry ISS grouping (12–39, 40–75)				
Year	0.975 (0.960–0.991)*	1.005 (0.963–1.049)	0.976 (0.962–0.990)*	
Constant	7.59e + 20 (2.00e + 07–2.88e + 34)*	0.0000228 (7.34e-43- 7.10e + 32)	3.19e + 20 (9.96e + 07–1.02e + 33)*	
*Statistical significance achieved at 5% level				

**Fig. 1:**
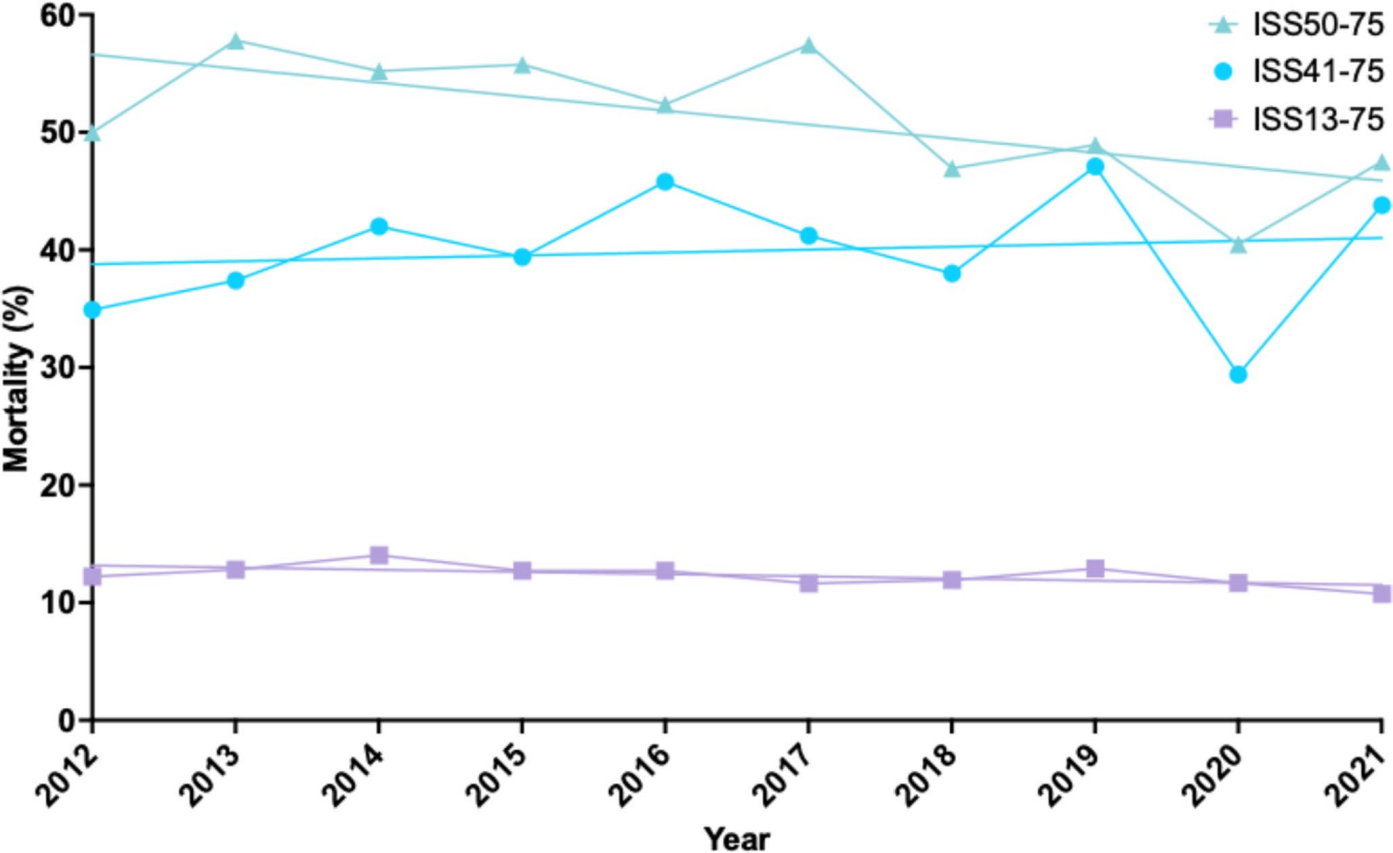
Mortality change over time

**Fig. 2:**
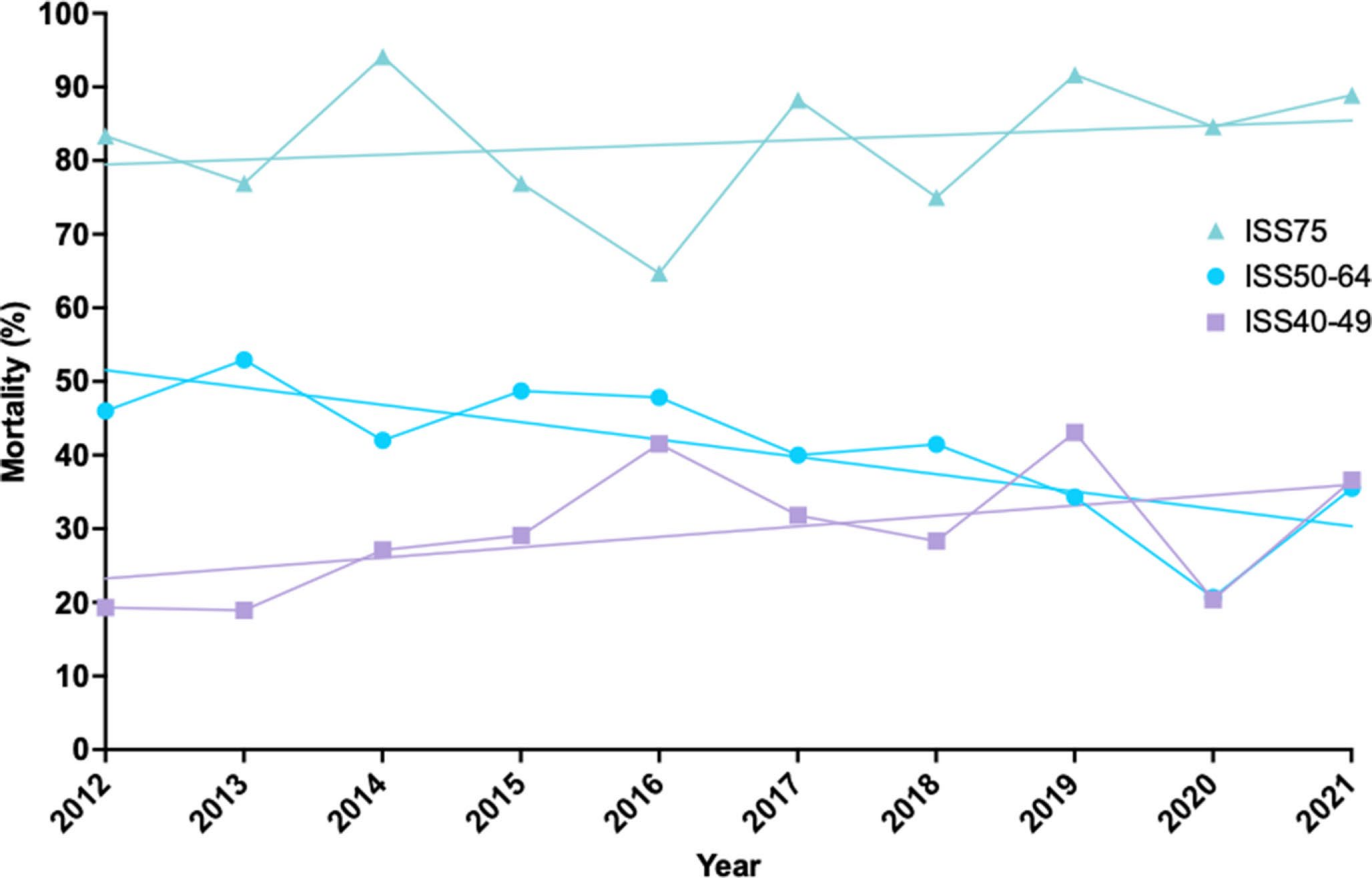
ISS40-75 subgroup mortality over time

Overall ISS13-75 mortality rate was 9.8%. The mortality rate improved over the study period (Table 1; Fig. 1). The lower mortality rate was driven by an increased survival in the more numerous, less severely injured ISS13-39 trauma patients while there was no improvement among ISS40-75 patients (Table 1). Post-hoc exploration of the ISS40-75 group visually, revealed three distinct subgroups with conflicting directions of significant change (Table 2; Fig. 2). ISS40-49 patients’ outcomes worsened, ISS50-74 patients’ outcomes improved, and ISS75 patients experienced no change (Table 2; Fig. 2).

**Table 2:** Outcome by ‘detailed’ ISS grouping

	Detailed ISS grouping				
	13–39	40–49	50–64	75	Total
Patients, n (%)	26,751 (96.0%)	584 (2.1%)	402 (1.4%)	125 (0.4%)	27,862 (100.0%)
Mortality, n (%)	2,291 (8.6%)	171 (29.3%)	170 (42.3%)	103 (82.4%)	2,735 (9.8%)
Logistic regression for Mortality by detailed ISS grouping (12–39, 40–49, 50–65,75)					
Year	0.975 (0.960–0.991)*	1.079 (1.012–1.150)*	0.913 (0.850 − 0.980)*	1.049 (0.878–1.252)	
Constant	7.59e + 20 (2.00e + 07–2.88e + 34)*	2.27e-67 (1.6e-123–3.25e-11)*	7.11e + 79 (4.52e + 17–1.1e + 142)*	1.46e-41 (8.4e-197 - 2.5e + 114)	
*Statistical significance achieved at 5% level					

## Discussion

Overall trauma mortality in this population is at an all-time low in developed trauma systems. This study reveals that these improvements have not been shared equally among patients with differing injury severities. The current research is the first to explore longitudinal change in high ISS subgroups and comparing them with the known improvements in the more numerous but less severely injured patients captured by a mature trauma system’s registry. Despite improving and world-standard major trauma mortality rates in this large trauma system, some groups of patients have a higher risk of inpatient mortality than they did ten years ago. The worsening of the ISS40-49 group’s mortality cancels out the improvement in the ISS50-74 group. This multi-centre retrospective study confirms the findings of a pilot single centre study which characterized the “critical polytrauma” group in the Australian registry setting [15]. We have demonstrated the practicality of capturing this rare, critically injured population in a multi-centre trauma registry.

There are several limitations to this study. This is a retrospective analysis of a prospectively collected trauma registry. Contributing to the registry is mandatory for major trauma centres and the registry uses multiple mechanisms to identify and record patients that were initially missed; however, this may still have occurred. The outcome measure, mortality, does not capture the reduction in quality of life experienced by some in the survivor group. The Injury Severity Score is an imperfect measure of mortality risk, with identical ISS values composed of different AIS triplets yielding considerably different mortality [21]. The ISS remains the most widely used risk adjustment system despite the proposal of alternatives [22].

The registry does not contain robust physiological data so no adjustment for physiological state was possible. This is a considerable limitation in a developed trauma system’s registry. The outcomes here may be a function of lack of adjustment for physiological derangement, rather than a true finding. Statistical adjustments for age and sex were not performed. This was a deliberate decision to demonstrate the utility of analysis of high ISS groups without adjustment, given our registry’s lack of physiological adjustment. The subgroup analysis of the ISS40-75 which led to the discovery of the divergence of mortality within, was not-prespecified. Pre-hospital mortality was not captured by the registry. Improvement of pre-hospital systems and resuscitation may be bringing patients who may have previously succumbed to their injuries on the roadside, to within reach of the trauma registry’s capture as an in-hospital death. It is not clear however why an improved prehospital service could have led to a worsening of the ISS40-49 group while simultaneously seeing improvement in the ISS50-74 group. The geography of our trauma system presents considerable challenges. Despite critical trauma being rare, our state has seven major trauma centres [16]. Three of these trauma centres are within a 10 km radius. Our study did not evaluate the effect of trauma volume on outcome however this has been demonstrated in other studies [23].

## Conclusion

Despite rightful celebration over continued improvements in headline survival rates [16], some specific groups of patients may be experiencing a worsening of outcomes. Prospective, comprehensive collection of high ISS subgroups should occur with reporting of them at an international level. ISS40-49 is identified as a group for targeted quality improvement.

Tables.

## Data Availability

No datasets were generated or analysed during the current study.
